# Analog Coding in Emerging Memory Systems

**DOI:** 10.1038/s41598-020-63723-z

**Published:** 2020-04-22

**Authors:** Ryan V. Zarcone, Jesse H. Engel, S. Burc Eryilmaz, Weier Wan, SangBum Kim, Matthew BrightSky, Chung Lam, Hsiang-Lan Lung, Bruno A. Olshausen, H. -S. Philip Wong

**Affiliations:** 1 Redwood Center for Theoretical Neuroscience, UC Berkeley, Berkeley, CA 94720 US; 2 Department of Electrical Engineering and Stanford SystemX Alliance, Stanford University, Stanford, CA 94305 US; 3 IBM Research, T.J. Watson Research Center, Yorktown Heights, NY 10598 US; 4 Macronix International Co., Ltd., Emerging Central Lab, 16 Li-Hsin Road, Hsinchu Science Park, Taiwan; 5 Google Brain, 1965 Charleston Rd., Mountain View, CA 94043 US

**Keywords:** Electrical and electronic engineering, Computational science, Information technology, Electronic devices

## Abstract

Exponential growth in data generation and large-scale data science has created an unprecedented need for inexpensive, low-power, low-latency, high-density information storage. This need has motivated significant research into multi-level memory devices that are capable of storing multiple bits of information per device. The memory state of these devices is intrinsically analog. Furthermore, much of the data they will store, along with the subsequent operations on the majority of this data, are all intrinsically analog-valued. Ironically though, in the current storage paradigm, both the devices and data are quantized for use with digital systems and digital error-correcting codes. Here, we recast the storage problem as a communication problem. This then allows us to use ideas from analog coding and show, using phase change memory as a prototypical multi-level storage technology, that analog-valued emerging memory devices can achieve higher capacities when paired with analog codes. Further, we show that storing analog signals directly through joint coding can achieve low distortion with reduced coding complexity. Specifically, by jointly optimizing for signal statistics, device statistics, and a distortion metric, we demonstrate that single-symbol analog codings can perform comparably to digital codings with asymptotically large code lengths. These results show that end-to-end analog memory systems have the potential to not only reach higher storage capacities than discrete systems but also to significantly lower coding complexity, leading to faster and more energy efficient data storage.

## Introduction

A paradigm shift in the type and quantity of storable information is currently underway. Internet-connected devices are projected to reach 50 billion, more than 6 devices per person, by the year 2020^1^. Much of the data produced by these devices, such as pixel intensities from cameras, sound recordings from microphones, and time-series from sensors, comes from signals that are intrinsically analog-valued or many-valued (>100 values) and ordinal. These signals are also highly redundant and compressible, with a large degree of their joint activity explained by a smaller number of factors than the intrinsic dimensionality of the signal. Furthermore, an increasing portion of the operations performed on the data are analog, with much of it being used for statistical inference or human perception (e.g. object-detection for images).

A concurrent paradigm shift is underway in the media we use to store data. Early storage media such as phonograph records and VCR tapes relied on perturbing an analog-valued state (wax height and magnetic polarization, respectively). Digital computation led to the popularity of binary storage representations that inhibit noise propagation and utilize the concurrently developed theories of binary error-correcting codes^2^. However, many emerging memory technologies have shifted back to analog-valued media to create multi-level devices that fill the need for inexpensive, high-density storage. MLC-Flash, Phase Change Memory (PCM), Resistive RAM (RRAM), and Conductive Bridge RAM (CBRAM) are all examples of technologies that have an analog state (threshold voltage or resistance) determined by the gate-charge, resistive amorphous capping region, and conducting filament, respectively^3,4^. One of the goals of many researchers and developers studying emerging memory technologies is to create a “Storage Class Memory”^5^. This is, essentially, a system of memory that combines the benefits of storage systems like HDDs (e.g. high density, long life) with the benefits of “high performance” systems like DRAM (e.g. fast access time). From this traditional perspective, the emerging memory device we will be considering – PCM – is approaching a Storage Class Memory system in that it has the ability to reach high densities (through 3D integration) with very fast access times (~100 ns).

The aforementioned trends of (i) analog data generation and operation and (ii) analog storage media development motivated us to investigate the use of analog coding in emerging memory systems. We examine here the potential for memory systems that utilize analog representations–analog-valued signals stored in analog-valued devices–to achieve higher capacity and reduced coding complexity, leading to reduced latency and increased energy efficiency.

To create a fair comparison between digital and analog systems, we need to compare their end-to-end performance on a storage task. Rate-distortion theory, introduced by Shannon in 1948, provides a common framework to evaluate both approaches, as it allows us to analyze the tradeoff between representation size and performance (i.e., rate and distortion, respectively, described in the following paragraphs)^6^.

**Figure 1:**
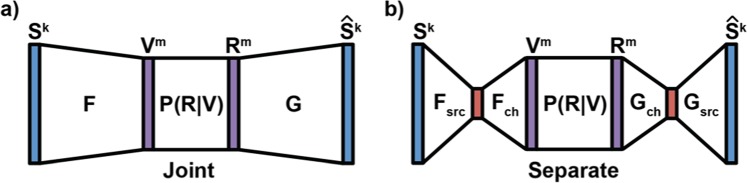
Illustration of Coding Strategies. The dimensionality of the signal is represented by the size of the bar. **(a)** Joint Coding. A k dimensional source, S^k^, is encoded with a function F into an m dimensional representation, V^m^. This is stored into analog devices and later read as the resistances R^m^. The characteristics P(R|V) are determined by a combination of the dynamics of both the memory elements and control circuitry. The resistances are then decoded with the function G into a reconstructed source signal (Ŝ^k^). **(b)** Separate Coding. Each encoding and decoding step is broken into separate source and channel procedures (F_src_, F_ch_, G_ch_, and G_src_, respectively). In discrete systems, source coding is usually applied in software (such as JPEG for images), and channel coding is applied in an on-chip memory controller. Such schemes can be optimal, but require asymptotically large blocklength to be so (k and m go to infinity, while k/m stays constant).

As diagrammed in Fig. 1a, the general coding framework can be set up as follows: a source signal, *S*^*k*^, of dimensionality *k*, is encoded (*F*) into a representation, *V*^*m*^, of dimensionality *m*. The encoded signal is then stored in memory devices, equivalent in the communications literature to passing through a noisy channel, characterized by *P(R*|*V)*, resulting in a noisy recalled signal, *R*^*m*^. A decoder (*G*) then creates an estimate of the original source, *Ŝ*^*k*^.

The channel noise characteristics, *P(R*|*V)*, uniquely determine the capacity of the channel (Eq. 1). Crucially, for memory storage, the noise distribution is determined by the dynamics of both the device response and of the memory control circuitry. For example, closed-loop memory controllers can increase channel capacities through multi-pulsed read-verify schemes^7^. Such schemes help increase capacity and compensate for device-to-device variations and temporal drift^8^, and can be described by information theoretic treatments without loss of generality^9^. For clarity, we restrict our current investigation to PCM devices controlled with a single read-write pulse, where *V* and *R* directly correspond to the write voltages and read resistances of *m* devices used to store *k* symbols of data.

The distortion (*d*) is determined by comparing the source and its reconstruction with a *task-relevant* metric. For example, if mean squared error (MSE) is the relevant metric, then $d=\frac{1}{N}{\sum }_{i=0}^{N}{({S}_{i}-{\hat{S}}_{i})}^{2}$, where *N* is the total number of examples. For media such as images, perceptual distortion metrics such as multi-scale structural similarity index (MSSIM) may be more appropriate^10^. For a given source, the rate (*r*, not to be confused with resistance) is given by the compression ratio *k/m*. For example, in a binary system with rate *r*, a source signal of *k* bits can be stored in *m* bits of memory and reliably recalled with a distortion *d*.

In contrast to the joint approach shown in Fig. 1a, Fig. 1b shows the traditional, separate approach to coding: encoding and decoding are each divided into separate coding procedures for the source and the channel (*F*_*src*_*, F*_*ch*_*, G*_*src*_*, G*_*ch*_). In this separate coding paradigm, source coding aims to remove statistical redundancy from the data. The resulting compressed representation requires fewer resources to store but is extremely fragile with respect to noise. Consequently, channel coding introduces redundancy, in the form of statistical dependencies, through an error-correcting code (ECC), typically with addition of parity bits dependent on data bits. Thus, if the channel code used is a graphical code, this enables the original source coded data to be decoded/inferred from the noisy version through a graphical model^11^.

Since small errors in a source coded signal can produce large distortions, sufficient redundancy must be added to ensure that no uncorrected errors remain, leaving source coding as the sole source of distortion. The degree of required redundancy (devices/bit) is determined by the inverse of the information capacity (*C*, bits/device) of the storage devices themselves, which is the maximum rate achievable with an optimal ECC. Thus, the capacity of an analog-valued memory device provides a fair means of comparison between digital and analog implementations. For each symbol produced by a source, a lower bound on *r(d)* (devices/symbol) for a separate-coded digital system is given by *r(d)* (bits/symbol) of the source coder divided by the capacity of the memory device (bits/device).

Shannon proved that errorless transmission of a source over a channel is only assured in the limit that the code word length, also known as the blocklength, approaches infinity^6^. Gastpar^12^ and Rimoldi^13^ shed new light on the problem by showing that the reason why separate codes often require large blocklengths is that they are effectively creating a deterministic channel, whereas a joint approach can keep the mapping random. More specifically, rather than trying to make the channel behavior asymptotically deterministic, joint coding attempts to map the statistics of the source through the stochasticity of the channel such as to produce a low average distortion. Under certain conditions, such mappings can achieve optimal *r(d)* with finite blocklength. Simple but common examples include the Gaussian source through a Gaussian channel with MSE distortion and the Bernoulli source through a binary symmetric channel with Hamming distance distortion. In both cases, the optimal solution is achieved by performing no coding at all (just simple scaling) and sending each symbol individually (blocklength of one)^12^. Further, even when the optimal solution is not reached, joint coding can significantly outperform separate coding schemes in the finite blocklength regime^14^. These examples demonstrate how matching the statistics of the source, channel, and cost can significantly increase the efficiency of reliable communication.

On the flip side, this attempt to create an effectively deterministic channel is why modern binary ECCs with rates that approach the Shannon capacity, such as LDPC codes^15^, often do so at the expense of large blocklengths (>1kB). While the degree of redundancy remains constant for large blocklengths, the expense of the decoder increases dramatically as it must perform inference on problems of higher and higher dimensionality. Even well-designed LDPC decoders with large blocklengths can consume significant power (~100 mW–1 W) and area (~10 mm^2^)^16,17^. As code lengths increase, so do latencies due to decoding, in one case rising from 20 us to 220 us for an increase in BCH (Bose-Chaudhuri-Hocquenghem) code length from 16B to 2048B^18^. While increasing the dimensionality, *k*, of the signal improves coding performance, it also requires reading from larger segments of memory at once and increases the complexity of the decoder circuitry, resulting in larger access latency and greater energy consumption. Furthermore, these separate digital systems typically lack a robustness to varying channel conditions: the performance saturates if the channel SNR increases beyond the value for which the system was designed and, in the other direction, the system does not “gracefully degrade”, like joint systems can, with decreasing channel SNR^19,20^.

Again, we can reformulate the problem of storing analog data on an emerging memory device as that of transmitting a discrete time, continuous alphabet source over a discrete time, additive noise channel. When thought of in this manner, we can then look at the general problem of analog coding in the field of communications and see what ideas can be brought to bear on this specific problem. This field has a rich history, with the original treatment of this problem dating back to Shannon^21^ and Kotelnikov^22^. Their proposed analog coding scheme was based on the use of space-filling curves. The use of space-filling curves was then significantly extended in the work of^23–30^. In these works, space-filling curves were investigated for the transmission of Gaussian sources over additive white Gaussian noise (AWGN) channels. These ideas, primarily using variants of the “Archimedean spiral”, were further extended by^31–34^ to the case of different kinds of channels. Following these, a major breakthrough was made in 2014^35^ when it was shown that instead of using parameterized spirals, a functional optimization approach could be used to achieve state-of-the-art performance on the problem of Gaussian sources over AWGN channels.

Here, inspired by the general success of optimization-based methods and utilizing ideas from the analog communications literature to frame the problem, we expand and build on our previous work^36^ and show that using analog codes with analog emerging memory devices can improve system performance over digital codes in both the separate coding and joint coding regimes. Using PCM as an example of analog-valued emerging memory, we demonstrate that separate coding systems can reach a higher rate of information transmission when paired with analog ECCs, as has been demonstrated in the literature for standard communications channels. In the joint coding regime, we show that analog codes can achieve *r(d)* performance comparable to digital codes, but at a lower complexity cost. Specifically, we examine the simple case of developing optimal mappings for storing analog values directly in the analog resistances of the PCM devices. For symbol-by-symbol storage of a Gaussian source with an MSE distortion metric, we find comparable *r(d)* performance to digital implementations employing optimal capacity-achieving channel coding. This surprising result is achieved with a blocklength of one, indicating the potential for even better performance with higher dimensional encodings utilizing the joint statistics of multidimensional sources. This work opens the door to further research into analog coding schemes and shows how concepts from analog coding can be applied to the problem of storing information on emerging memory systems, with the potential to outperform digital approaches. We have seen in previous work^37,38^ that certain analog coding schemes for emerging memory devices can empirically outperform digital ones, and this current work helps to provide an explanation as to why.

## Background

**Figure 2:**
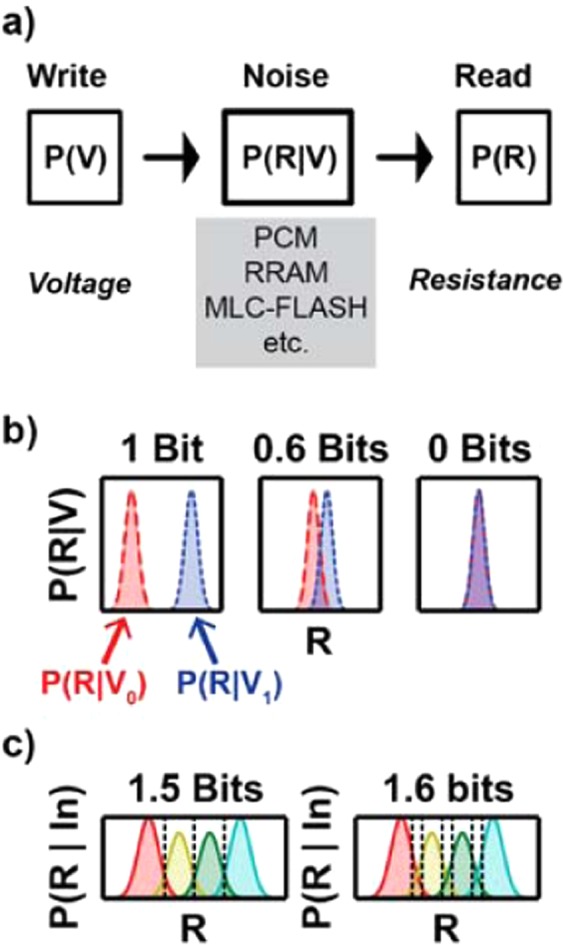
Storage as a Communication Problem. (**a**) The capacity (Eq. 1) is determined by the ability to infer a write voltage pulse distribution P(V) from the read resistance distribution P(R). The conditional dependence between the two, P(R|V), is unique for each technology and pulsing scheme. While we chose a particular technology (and therefore a particular P(R|V)) to illustrate the main points of the paper, the framework could be applied to any emerging memory device (e.g. RRAM, STT-MRAM). **(b)** Example of two state system, with input voltages V_0_ and V_1_. If there is no overlap between the output distributions (left), the capacity is log_2_(# write states) = 1 bit. For complete overlap (right), the capacity is 0 bits, as it is impossible to infer anything about the inputs from reading the outputs. For partial overlap (center), redundancy needs to be added to the signal to correctly infer the inputs, reducing the effective bits/device. **(c)** ‘Soft information’ increases capacity. For a given number of write states (4), the capacity increases with number of read states (4 left, 7 right). The read resistance is discretized into bins separated by black dotted lines. Even though there are only 4 input states, the extra read states increase the capacity of the system by providing ‘soft information’, the degree of belief that a read value belongs to each write value. Further read states provide greater granularity of belief values, allowing for easier inference of the input values. Analog codes, such as artificial neural networks, can use the actual resistance values, intrinsically benefiting from high granularity.

To measure the capacity of analog-valued PCM devices, we recast the storage problem as a communication problem (Fig. 2a). The devices are perturbed by voltage pulses of different magnitudes drawn from the input probability distribution P(V). These pulses modulate a device’s resistance, resulting in an output resistance distribution ${\rm{P}}({\rm{R}})=\int {\rm{P}}({\rm{R}}|{\rm{V}}){\rm{P}}({\rm{V}}){\rm{dV}}$. The conditional dependence of the read resistance on the write voltage, P(R|V), defines the noisy channel through which information must be communicated. In the limit of an infinite blocklength code, the capacity is the number of bits of information per use of the device that can be communicated without error^6^. It is equivalently given by the maximum mutual information between the input and output distributions,

1 $$C=ma{x}_{P(V)}\sum _{V}P(V)P(R|V)lo{g}_{2}\frac{P(R|V)}{P(R)}$$

The capacity achieving input distribution given by Eq. 1 exhibits an optimal tradeoff between having as many input states as possible and having as little overlap in the output distributions as possible. As a simple example, consider a two-state system shown in Fig. 2b, with input voltages V_0_ and V_1_. If there is no overlap between the output distributions P(R|V_0_)P(V_0_) and P(R|V_1_)P(V_1_) (left panel), the capacity is the log (base two) of the number of input states (1 bit). If there is complete overlap (right panel), the capacity is 0 bits, as it is impossible to infer V from R. For cases of partial overlap, redundancy needs to be added to the signal to correctly infer the inputs, reducing the effective bits/device (center panel). Increasing the number of read states can also increase the capacity for a given number of write states (Fig. 2c). Practically, having more read states than write states gives additional information in the form of greyscale belief as to which input state they belong. This ‘soft information’ is currently used by MLC-Flash LDPC decoders to improve inference during belief propagation decoding^16^. Intuitively, analog representations that use the resistance values directly can have higher capacity because they operate at higher granularity (ultimately limited by circuit noise).

### Device operation

**Figure 3:**
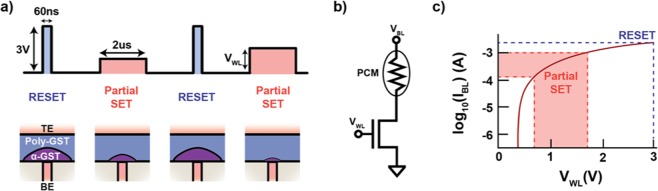
Device Operation. (**a**) Illustration of the pulsing scheme. Between each measurement, the cell is consistently “RESET” into the high resistance state via a short current pulse that melts the region above the bottom electrode (BE) and quickly cools to form a resistive amorphous cap (left). The cell is then “Partial SET” to a lower resistance with a long wordline (V_WL_) voltage pulse that anneals the amorphous cap region (second from the left). Larger voltage pulses (larger V_WL_) create smaller amorphous caps (far right) and thus yield lower resistances. (**b)** Circuit diagram of a single device within the 10 × 10 PCM array. Devices in the array are individually addressed by applying voltage V_WL_ to the gate of the access transistor. PCM resistance is modulated by current flow whose magnitude is controlled via applying a large bitline voltage (V_BL_ = 3 V) and pulsing the wordline. **(c)** I-V diagram showing the relationship (red curve) between V_WL_ and current flowing through the device. Larger V_WL_ create larger currents through the device.

Device operation is carried out in the following manner: as diagrammed in Fig. 3a, a PCM material can either be in a conductive crystalline structure (Poly GST, light blue) or a resistive amorphous structure (α-GST “cap”, light purple). The resistance of the PCM cell is determined by the size and structure of the resistive amorphous cap, with larger caps yielding higher resistances. The resistance is set to a high resistance state by a short current pulse (Fig. 3a, top panel, left-most light blue pulse) that first melts the film through Joule heating. The short pulse then quickly quenches the material, causing it to form a resistive amorphous cap. The resistance can be decreased by slowly passing a lower current pulse (Fig. 3a top panel, second pulse from the left, light red) that heats the material above its crystallization temperature, annealing the amorphous cap, allowing some of the crystalline structure to slowly reform while cooling. Different resistance levels can then be achieved by applying different magnitudes of current pulses (e.g. the two different light red pulses in Fig. 3a, top panel), resulting in different volumes of resistive amorphous and conducting crystalline phases^3^. In general, closed form mathematical models do not do a good job of describing the dynamics of PCM devices. To describe the dynamics of these devices requires highly detailed finite-element modeling (e.g. COMSOL^39^), and even this is not very accurate in most circumstances because there are intricate interactions between the electric field, current, and temperature-dependent conductivity of the GST that all must be simulated self-consistently^40,41^. For these reasons, we construct a numerical model of these devices for our study based on empirical measurements. We describe the data collection and model in the following paragraphs.

For this study, we performed pulsed resistance measurements of seven different devices (Fig. 3b, circuit diagram for one such device) on a 100-device PCM array. Details of fabrication and characterization of these arrays can be found in previous reports^42–44^. Each cell is equipped with its own access transistor (Fig. 3b) to prevent cross-talk and sneak paths in the array. Current flowing through the cell is controlled by the gate ‘word line’ voltage (V_WL_), which we pulse to control the shape and magnitude of current pulses. To ensure independence between pulses, we first ‘RESET’ the cell to a high resistance state with a short melting pulse. We then apply a longer variable-magnitude ‘Partial SET’ pulse to decrease the resistance of the cell. This facilitates analysis by constraining device behavior to a memoryless zeroth-order Markov process. For example, let us say that at t = 1 second the value of V_WL_ = 1.0 Volts was applied across the device. This will result in a resistance that is drawn from the conditional distribution P(R|V_WL_ = 1.0). Now because of the way the device is being pulsed, this conditional distribution will be the same regardless of what the previous resistance value was. I.e. if the process had memory, P(R|V_WL_ = 1.0) could be different depending on what R at t = 0 seconds was. But since the process does not have memory, P(R|V_WL_ = 1.0) will always be the same [Note: It should be noted that a digital system would need to apply a similar pulse scheme if it was to achieve greater than 1 bit of information transmission across the channel.]

## Results

### Capacity

As mentioned earlier, device-to-device variation and resistance drift will have a dramatic effect on the capacity of PCM arrays. The channel noise characteristics are determined by both the memory controller and memory devices, and indeed multi-pulse read-verify control schemes have proven effective at achieving high capacities despite device-to-device variation and resistance drift^7,8^. As a compromise between simplicity and realism, we ignore drift in our current treatment and approximate a simple memory controller to measure the responses of seven different devices on this 100-device array.

For each of the seven different devices, we first collect 120 trials at each voltage level. With seven devices, this corresponds to 840 points at each voltage level. For this study, we used 100 voltage levels. To then calculate a continuous probability density for P(R|V_WL_), we first performed Gaussian kernel density estimation (KDE) of the distribution of resistances resulting from each voltage level. That is, for each of the 100 voltage levels, v, a Gaussian KDE of P(R|V_WL_ = v) was made from the 840 measured points. To estimate P(R|V_WL_ = v) for v’s that were not measured, we simply interpolate between the two closest P(R|V_WL_ = v) that we have measurements for.

**Figure 4:**
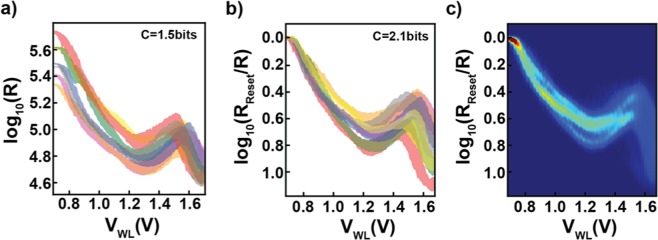
Measuring P(R|V). (**a**) Plot of the data collected for 100 different values of voltage pulse V_WL_ for seven different devices shown superimposed (each in a different color). Rather than plotting the 84,000 raw data points collected, we instead plot filled-in curves where the top and bottom boundary of each curve are +/− one standard deviation from the mean of R, respectively. This gives a better sense of where most of the raw data is concentrated for each of the seven devices. Values of V_WL_ between those collected (and the corresponding +/− standard deviations) are linearly interpolated. Lumping all seven devices together into a single ‘virtual device’ yields a capacity of 1.54bits. Notice that the different devices exhibit qualitatively similar behavior but with slightly offset RESET resistances and slopes of annealing. **(b**) The same as in (a), but now with normalized RESET resistances (achievable via a simple single-pulse memory controller). This normalized data yields a capacity of 2.08 bits. **(c)** Heat map of the conditional distribution, P(R|V_WL_), estimated using Gaussian KDE and linear interpolation on the normalized data points.

Plotted in Fig. 4a are one standard deviation above and below the mean for each of the seven devices measured (interpolating between the 100 measured v’s to get the smooth estimates shown). As can be seen, the different devices exhibit qualitatively similar behavior with slightly offset RESET resistances and slopes of annealing. We approximate the very simple single-pulse memory controller, mentioned above, by normalizing the RESET resistances between devices through measuring the relative resistance (to the RESET resistance) rather than the absolute resistance, which can be accomplished with two resistance reads and a divide operation.

Another way of thinking about this with Fig. 4a in mind is that we’re simply applying a vertical shift to each of the curves so that their left most points align, yielding the curves seen in Fig. 4b. Now, in this new space, the relative resistance value of a point is the logarithmic ratio between R_Reset_ and the measured resistance, i.e. log_10_(R_RESET_/R) (y-axis, Fig. 4b). Thus, all the points to the farthest left are centered around 0.0, by construction (as this is log_10_(R_Reset_/R_Reset_)), while all the points that are, for example, 10 times smaller than R_Reset_ are at 1.0.

The effect of this normalization on the actual measured resistances is that it brings all of the points for each voltage closer together. Thus, the seven curves described previously from Fig. 4a now lie on top of each other (Fig. 4b) as the seven sets of 120 points that yielded each curve are now much closer together. Finally, when the Gaussian KDE (with interpolation) is done on this new, renormalized set of points, the P(R|V_WL_) [Note: More precisely, this is P(log_10_(R_RESET_/R)|V_WL_), but for brevity and clarity we’ll refer to log_10_(R_RESET_/R) simply as R in the text.] shown in Fig. 4c results [Note: Raw data and the code to reproduce the parameters of this Gaussian KDE can be found at https://github.com/rzarcone/Analog_Coding_Final].

From this empirically obtained P(R|V_WL_) we then solve for the capacity-achieving input distribution, P_cap_(V_WL_), using the Blahut-Arimoto algorithm^2^. The effect of the simple single-pulse memory controller is that increases the capacity of the channel from 1.54 bits to 2.08 bits (for reference, the average capacity for any individual device is ~2.5 bits). While more realistic arrays would contain more variation and drift, they would typically also be paired with more powerful controllers, regardless of the coding scheme (either analog or digital). Thus, as they would affect both schemes, we will not factor them into our central comparison of analog vs. digital coding for emerging memory devices. Furthermore, opportunities exist to jointly learn the behavior of the controller with the encoder and decoder – e.g. with a neural network – which is in fact an avenue we have explored in another paper^37^.

**Figure 5:**
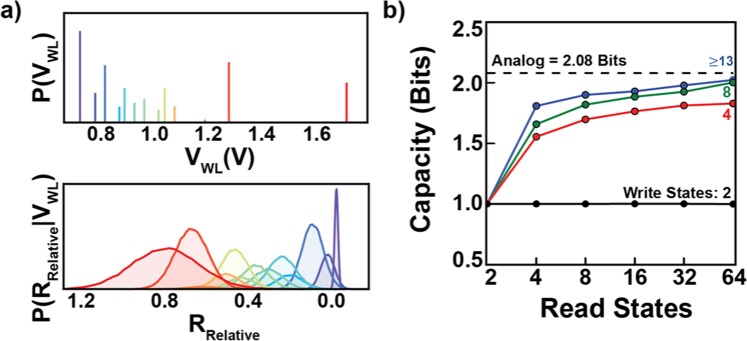
Calculating Channel Capacity. (**a**) Capacity achieving input distribution, P(V_WL_), and corresponding output distributions, P(R_Relative_|V_WL_), shown for each of the discrete levels in P(V_WL_) (each in a different color)[Note: Here we have replaced log_10_(R_Reset_/R) with R_Relative_ to not clutter the figures.]. The optimal input distribution contains 13 discrete levels, spaced so as to minimize overlap in the output distributions (additional input states beyond this can be used, but they do not increase the capacity). Note that even though the input distribution is over a finite number of states, the output distribution covers the full analog range of R. **(b)** Discrete capacity as a function of the number of read and write states. Limited by the number of write states, capacity increases with the number of read states due to the creation of ‘soft information’. For more than 13 write states, the discrete capacity asymptotes to the analog capacity as the number of read states increases. Thus, error-correcting codes that utilize analog circuits and the actual cell resistance values (such as those in an artificial neural network) can achieve the highest possible rates.

As the Blahut-Arimoto algorithm technically only applies to discrete distributions, we use a discrete approximation to the channel, finely discretizing the P(R|V_WL_) that we calculated as discussed above. Our criterion for sufficient discretization is that the capacity no longer increases with the addition of more states (e.g. >1000 states). As the goal is to optimize the tradeoff between the number of output states and these states’ overlap (Fig. 5a), the number of nonzero values in P_cap_(V_WL_) eventually saturates. That is, beyond a certain number of discrete input states, the addition of more states does not increase the mutual information (if the full analog output range is used). For this channel, saturation is reached at 13 states [Note: Non-uniform input state probabilities like those here are difficult to achieve for many applications, but setting them equal only decreases the channel capacity by ~5%]. Note that the output distributions for these states partially overlap and cover the full range of R (Fig. 5a, bottom panel), meaning that higher capacity can only be achieved if the full analog output range is used (as opposed to the traditional discrete case where there is no overlap). Put another way, any decoding algorithm that is to achieve the channel capacity must perform inference over the entire analog range of R.

As mentioned previously, the capacity is the maximum possible rate of information transmission over a given channel. To gain a better understanding of the effect of discretization on the rate of information transmission, we examine the case where there are a finite number of read and write states. This analysis is done to illustrate and make quantitative, for the case of the device we’re considering, the idea of “soft information” presented in Fig. 2. We model this discretization behavior by marginalizing the continuous probability P(R|V_WL_) between discrete read levels, creating a reduced discrete channel. We then use simulated annealing to search for optimal values of the read levels to maximize the rate of information transmission. Figure 5b demonstrates how the capacity of this reduced channel increases with the number of read levels for a given number of write levels. In each case, the capacity monotonically asymptotes as the number of read levels increases, corresponding to the case of an analog read circuit. As the number of allowed input levels increases above 13, the optimal number for the full analog channel, the discrete capacity asymptotes to the analog capacity of 2.08 bits. Put another way, the maximum possible rate of information transmission is only possible in the limit that the full analog range for the output is used. As analog systems inherently operate over the full continuous range of values, it is possible for them to achieve the capacity. This use of the full continuous output range can be thought of as a necessary condition to achieve the maximum possible rate of information transmission over the channel. Of course, developing the specific code that achieves this maximum information transmission is an unsolved problem. I.e. the capacity tells you what the upper bound is, but not how to achieve it. This discretization analysis shows that it is necessary to consider the full continuous range of outputs to achieve this bound, but it too does not show you how to achieve it. Thus, if the goal is to perform separate source and channel coding, say, to allow the channel coder to work with data from a wider range of sources, then analog channel coding schemes have the ability to outperform digital ones.

### Joint coding

Finally, we explore an approach to directly store analog-valued information in the resistance values of the PCM devices. While many interesting opportunities exist for exploiting the rich statistics of natural signals, for this preliminary study, we restrain ourselves to the canonical case of a single Gaussian source signal, P(S), with an MSE distortion metric. The most naïve approach to store analog values would be to employ no coding and directly map source values, S, into voltages and directly provide reconstructions, Ŝ, from resistances. Such a naïve approach leads to an “effective channel” (conditional probability distribution of reconstruction, P(Ŝ|S)) identical to the original channel, P(R|V_WL_). While this is the ideal approach for a Gaussian source with Gaussian channel and MSE distortion^12^, the nonlinear PCM device is far from a Gaussian channel. Correspondingly, the naïve implementation exhibits a low SNR (< 0 dB) at a rate of 1 device/symbol.

Given that we are working with a Gaussian source and MSE distortion, we would like a parameterized model to learn an encoding and decoding function that minimizes the average cost. We start with a parameterized model

2 $$V=F(S)$$

3 $$\hat{S}=G(R(V))$$

where F and G are nonlinear mappings corresponding to the encoder and decoder, respectively. We then solve numerically for the mappings that minimize the distortion,

4 $${F}^{\ast },{G}^{\ast }=argmi{n}_{F,G}D=argmi{n}_{F,G}\int \int ({(\hat{S}-S)}^{2}P(\hat{S}|S)P(S)d\hat{S}\,dS$$

The encoding and decoding functions are parameterized by a sum of un-normalized Gaussians. Specifically, F(.) and G(.) are described by

5 $${y}_{\alpha }(x)=\mathop{\sum }\limits_{i=1}^{N}{y}_{{\alpha }_{i}}K\left(\frac{x-{x}_{i}}{\sigma }\right)$$

6 $$K\left(\,\frac{x-{x}_{i}}{\sigma }\,\right)=\frac{{e}^{\frac{-{(x-{x}_{i})}^{2}}{2{\sigma }^{2}}}}{\sqrt{2\pi {\sigma }^{2}}}$$

Here, the Gaussian centers, {x_i_}, as well as their widths, $\sigma$, are fixed, and the only variables to be adapted are the weights of each Gaussian, {${y}_{{\alpha }_{i}}$} [Note: see appendix for details on the fixed parameters]. Ultimately, two different ${y}_{\alpha }$ are learned, one for the encoder and one for the decoder. The {${y}_{{\alpha }_{i}}$} for each of these are learned by minimizing Eq. (4) via gradient descent. A sum of Gaussians is chosen because they are smooth (with well-behaved derivatives for the gradient-based optimization), are easily parameterizable, have support over the whole region considered, and are highly non-linear and thus, when combined, able to approximate a large class of functions.

**Figure 6:**
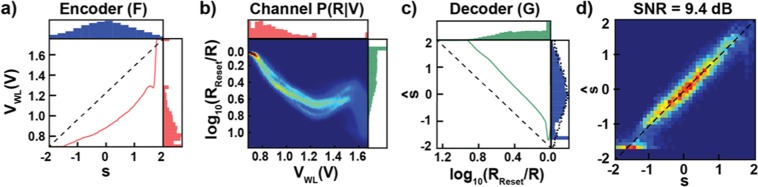
Optimal joint coding of a Gaussian source. (**a**) Samples, S, distributed according to a Gaussian (blue distribution, top) are mapped through the learned encoding function (red line). For reference, a linear mapping is shown with a dashed line. This encoding transforms the Gaussian distribution into a highly non-Gaussian distribution over V_WL_ (red distribution, right). Encouragingly, this distribution qualitatively matches the capacity achieving source distribution, P_cap_(V_WL_) (a quantitative comparison is difficult as the former has compact support over different regions while the latter has finite support). **(b)** Samples, V_WL_, distributed according to P(V_WL_) (red distribution, top), are then stochastically mapped through the channel (heat map in center), resulting in a distribution over resistances, P(R) (green distribution, right). **(c)** Samples passed through the channel, R, are then sent through the decoder, G (green line), which transforms them into estimates of the original input, Ŝ (again, a linear mapping is shown with a dashed line). This results in a distribution over reconstructed samples, P(Ŝ) (blue distribution, right), which is very close to the original Gaussian distribution (indicated with a dashed line). **(d)** Heatmap demonstrating how samples of S are transformed through the whole pipeline, resulting in reconstructions Ŝ. Note that as the majority of the mass for S is between [−1, 1], this is where the encoding/decoding functions learn to do the best.

**Figure 7:**
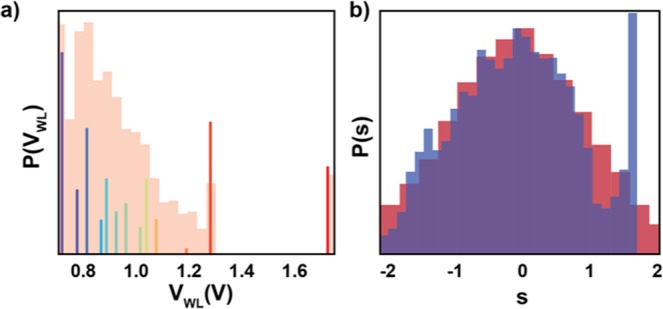
Distributions from learned mapping. (**a**) Reproduction from Fig. 5a of the capacity achieving source distribution, rainbow, overlaid on the distribution produced by the learned encoder (the same distribution as shown in Fig. 6a, red, and Fig. 6b, red). Notice how the learned mapping captures most of the features of the capacity achieving distribution: largest concentration of mass around the minimum value of V_WL_, with a taper until a spike around 1.3, followed by zero mass until one final spike at the maximum value of V_WL_. **(b)** Reproduction from Fig. 6c of the mapped output distribution, blue, overlaid on the source distribution, red. Notice again how the learned mapping does the best job of reconstructing the input in the region [−1, 1], where the majority of the mass is.

The results of this minimization are presented in Fig. 6. Here, the Gaussian distributed variable, S (blue distribution, top of Fig. 6a), is passed through the learned encoding function F (red line, Fig. 6a). That is, each sample from P(S) is mapped, deterministically, through F into a specific voltage, V_WL_. For example, a sample of S = 0.0 is mapped to approximately 0.9 V. This mapping transforms the original Gaussian distribution into a highly non-Gaussian distribution (red distribution, Fig. 6a right and Fig. 6b, top). Interestingly, and encouragingly, this distribution is qualitatively similar to P_cap_(V_WL_) (Fig. 5a). For an easier comparison between the two we have taken the resulting P(V_WL_) and overlaid P_cap_(V_WL_) (Fig. 7a).[Note: It is difficult to make a quantitative comparison here as the capacity achieving source distribution is over a finite number of elements whereas this distribution has compact support in different sections.] It is also interesting that the parameterization for the encoding function, the sum of Gaussians, was chosen for its flexibility in function approximation, and not hand-designed to try and approach P_cap_(V_WL_). Thus, the fact that algorithm was able to learn a mapping that produced a distribution that approaches P_cap_(V_WL_) is support for the algorithm’s generality.

The encoded value then gets stochastically mapped through the channel. That is, each sample voltage results in a draw from the conditional distribution given by P(R|V_WL_). For example, V_WL_(S = 0.0) results in a draw from P(R|V_WL_=0.9). The distribution of resistances that results from this mapping is again highly non-Gaussian (green distribution Fig. 6b, right and Fig. 6c, top) [Note: Note that the encoder learns to map a large amount of mass in the region of the channel where input-output pairs are easiest to distinguish (red portion of histogram, where the noise is smallest) and none in the “indistinguishable” region of the channel (region with positive slope, approximately 1.2–1.6 V).] Finally, the read-out resistance is passed through the learned decoding function, G (green line, Fig. 6c). The resulting distribution for Ŝ is very close to the original Gaussian (with the target distribution for S given by the dashed line). For an easier comparison between these distributions we have also taken the original P(S) and overlaid P(Ŝ) (Fig. 7b).

To quantitatively assess the performance of this approach, we can draw many samples from P(S) and check what the corresponding Ŝ is. This creates an ‘effective channel’, P(Ŝ|S) (Fig. 6d). This analysis shows that samples from P(S) are mapped through the channel and recovered by Ŝ with a small amount of scatter around the straight dashed line (corresponding to Ŝ = S), yielding an SNR of 9.4 dB. Note how the region between [−1,1], where the majority of S’s mass is, is where the encoder and decoder learn to do the best job of transmitting the signal over the channel. This is a consequence of the MSE distortion metric: the encoder and decoder are penalized the most in this region and thus devote their resources to performing good reconstructions here.

As another point of comparison, we can look at a quantity from the analog coding literature: the Optimal Performance Theoretically Attainable (OPTA). This quantity is obtained by equating the channel capacity to the rate distortion function of the source considered, and then solving for the resulting minimum achievable distortion (usually in decibels). Thus, for the Gaussian source and the channel being considered we have:

7 $$\frac{1}{2}lo{g}_{2}\left(\frac{{\sigma }_{s}^{2}}{D}\right)=2.08\,bits$$

This implies that the OPTA is 12.5 dB (3.1 dB from our method). While this is a useful quantity to consider as it provides an upper bound to the performance, we prefer instead to think about the effective number of bits transmitted across the device (obtainable via a similar manipulation as above, but solving for rate instead of distortion). From this perspective, we think that the most surprising and important result of this work is that even with this very simple encoding/decoding scheme (amounting to a single dimensional lookup table), joint coding with these devices achieves a similar SNR to separate coding schemes of high complexity. Specifically, this joint method performs as well as a separate system that first compresses strings of samples from the Gaussian to 1.56 bits per sample (e.g. through vector quantization) and then uses a hypothetical channel coder capable of getting 1.56 bits of errorless communication across the device. Said channel coder is “hypothetical” in that there does not currently exist a channel coder capable of achieving 1.56 bits across the device. In reality, the design of channel coders for specific channels is a challenging task. For example, though the capacity of the standard AWGN was worked out by Shannon over 70 years ago^6^, it wasn’t until the 1990s that a class of codes was created that could approach the capacity of these channels^15^.

Though a channel coder for the separate coding scheme does not exist for these channels, we can assume one does for the purposes of a comparison. We can then further quantify the benefits of our approach by comparing the energy and latency required to perform the above storage task with separate vs. joint coding schemes. For the comparison, an ‘8KB’ PCM array is used, with both separate and joint coding schemes implemented in Samsung 28 nm technology. As discussed above, the separate approach requires the use of digital error-correcting codes. These codes require large blocklengths for even modest code rates (e.g., ~0.5). The current state of the art – both in terms of performance and efficiency – are LDPC codes. Thus, we use these as the hypothetical channel coder for the comparison. An LDPC code with good decoding performance typically requires a blocklength of ~2000 at a code rate of ~0.5 (with significantly longer blocklengths for higher rates)^45^. The decoding step alone for this requires ~44 pJ/sample. For this storage task, the total energy expenditure for a separate scheme with an LDPC code is ~128 pJ/sample. Furthermore, the LDPC decoder requires 10 (or more) iterations to converge. By comparison, a joint coding scheme, achieving the same SNR (i.e. at the same distance from the OPTA, described previously), implemented using a simple lookup table requires only ~40 pJ/sample and a single iteration. The decrease in required energy and latency come mainly from (i) the elimination of the LDPC coder – requiring long blocklengths – and (ii) the reduction in PCM write operations – 2 writes/sample → 1 write/sample – due to the higher coding rate achievable with joint coding in the small blocklength regime (via the intrinsic error-correction provided by the learned encoding/decoding functions).

While we have only examined the case of single dimensional encodings here, the design landscape for analog encoders/decoders is rich for higher dimensional signals and other compression ratios, making it a promising area for the development of coding circuitry that is low in energy and latency. Lastly, we should emphasize that because the joint framework we proposed is adaptive, it can learn to store different types of analog data on different types of channels, as diagramed in Fig. 2 (e.g., RRAM, MRAM, or other PCM devices).

## Discussion

We have explored a new approach for storing analog-valued media in emerging analog-valued devices showing that analog coding strategies have the potential to create robust storage with relatively low complexity. Designing systems to match the statistics of the source, channel, and task-at-hand provides unique opportunities not captured by systems that try to enforce determinism. However, there is no free lunch, and what such systems gain in efficiency they likely lose in generality.

Universal memory design has been a great boon to the development of digital technology and designing memory systems for specific tasks has many unexplored risks in terms of backward compatibility. That said, with the rise of machine learning applications, the nature of computation is changing, and many of the data-centric applications of the present and future do not require perfect data retrieval. With the data deluge faced by internet companies, it is often not the storage of data that is important, but the storage of information which performs well when retrieved for human or statistical machine inference (e.g., perception of audio and video). While losing some generality, representations that reduce the redundancy of signals to match the statistics of storage media and applications can be more efficient than universal representations.

It is interesting to note that from the perspective of neuroscience, neural systems are faced with a very similar task. Given the statistical redundancy of natural stimuli and the stochasticity of perception and signal transmission, efficient coding strategies must be devised to transmit compressed representations of stimuli that are intrinsically robust to neural noise and resource efficient^46,47^.

Finally, the results presented here represent only a single model applied to a single type of signal and device. While we only looked at one type of signal and device for illustrative purposes, the methods we used here are very general and can be applied to a wide range of devices and signals. The future design space to explore in this field is extremely rich.

From the experimental perspective, other devices (RRAM, CBRAM, and MLC-Flash) can exhibit dramatically different behavior. Furthermore, various pulsing schemes can produce different types of statistics. For example, iterative pulsing introduces rich conditional dependencies through time that are not currently exploited by multi-bit memory systems and present an interesting opportunity from the perspective of modeling. Cell-to-cell dependencies within the array are also a fact of life that must usually be avoided through hand-engineered systems. Measuring these statistical dependencies can enable codes to be adaptive to the flaws and statistics of individual chips, both at the factory during test time and over the lifetime of use, improving the reliability and uniformity of performance.

From an algorithmic perspective, many powerful analog-valued high-dimensional models such as artificial neural networks exist, leaving a rich space to explore for learning high-dimensional parameterized encodings and compression. Since such models have been able to successfully utilize the statistics of natural media such as images^48^ and sound^49^, they would seem well-suited for encoding such media in analog-valued devices. While these neural network methods have been successfully applied to the problem of source coding^50,51^, there has still yet to be a thorough treatment of the problem for doing both source and channel coding with emerging memory devices, and we think this is an exciting open area for research. Our own initial work in this direction^37,38^ has shown that a joint approach with neural networks can outperform traditional separate approaches when storing images on an array of emerging memory devices. In a related work^52^, researchers have looked at the problem of using neural networks for source and channel coding with standard channels, such as the Gaussian and Rayleigh channels. As mentioned above, many of the concepts we have discussed here are regularly applied to analog coding problems in the communications literature, typically in the context of communicating over standard channels. Our focus in this work has been on bringing these ideas into the problem of storage with emerging memory devices.

Efficient integrated circuit implementations of such circuits are also an active area of investigation^53^, lending further weight to the idea of analog-valued ECCs that can learn on-chip dependencies and adapt to nonstationary statistics as devices age. Another active area of research is in using analog-valued variable resistors as synaptic elements in such neural circuits^54–56^, leading to the possibility that storage and error-correction could someday be integrated within a single memory substrate.

## Methods

### Device operation

Pulsed resistance experiments were conducted using a Cascade probe station, measuring 1T1R (one transistor per resistance element) PCM arrays with approximately 40 nm Bottom Electrode (BE) contact diameter. Details on device fabrication and characterization of these arrays can be found in^42–44^. Wafers of arrays, provided by IBM, were contacted by a custom probe card and connected to a Keithley 700B Switch Matrix, enabling access to 100 unique devices. Voltage pulses were produced by an Agilent 81110 A Pulse Generator. ‘RESET’ pulses applied 3 V to bitline and wordline with a 5 ns rise time, 60 ns width, and 5 ns fall time. ‘Partial SET’ pulses applied 3 V to the bitline and a variable voltage to the wordline with 500 ns rise time, 2000ns width, and 500 ns tail. Cell resistances were measured with an Agilent 4155 C Semiconductor Parameter Analyzer, applying 3 V to the wordline and 0.1 V to the bitline. All equipment was controlled via custom Python software utilizing PyVisa and wxPython (available at http://www.github.com/jesseengel/PythonProbestation).

### Capacity comparison

#### Estimating P(R|V)

Estimates of P(R|V) were calculated from experimental data using Gaussian kernel density estimation (KDE) and linear interpolation, implemented using SciPy. Python code for this is available at https://github.com/rzarcone/Analog_Coding_Final.

### Calculating capacity

Capacity calculations and rate-distortion simulations were conducted in Python using NumPy. The implementation of the Blahut-Arimoto algorithm was adapted from MATLAB code by Kenneth Shum (http://home.ie.cuhk.edu.hk/~wkshum/wordpress/p=825). Code for this Python implementation is available at https://github.com/rzarcone/Analog_Coding_Final.

### Capacity for finite states

For examining the case of a finite number of read and write states, we employ a basin hoping search strategy, as outlined in^57^. Python code for this is available at https://github.com/rzarcone/Analog_Coding_Final

### Joint coding

#### Learning encoding/decoding functions

The encoding and decoding functions are parameterized by a sum of unnormalized Gaussians, as described in the main text. The fixed parameters, {x_i_}, $\sigma$, and N, are selected via a standard grid-search method. That is, an initial guess for each of these is chosen. The optimization is then done with these parameters resulting in a given performance level. The parameters are then modified, and the optimization is carried out again. The parameters that result in the best performance are then used in the final encoding/decoding functions. The objective for learning the variable parameters, the weights of these Gaussians, is minimized using Adam^58^, a version of gradient decent.

In order to be able to take derivatives through the stochastic devices during training, we use a version of the reparameterization trick^59^. That is, the numerical model described in the Capacity section of the main text is further simplified (only during training, but not during the actual evaluation) to be conditionally Gaussian, where the mean and variance of each Gaussian depends, highly nonlinear, on the voltage applied. Thus,

8 $$R(V)=\mu (V)+\sigma (V)\cdot \varepsilon ,\,\varepsilon  \sim N(0,1)$$

The functions describing how the mean and variance of the conditional resistance distribution change with V, $\mu (V)$ and $\sigma (V)$, respectively, are simply calculated from the measured mean and variance at each of the voltages probed, and then interpolated between these. Raw data and the parameters for the $\mu (V)$ and $\sigma (V)$ models can be found at https://github.com/rzarcone/Analog_Coding_Final.

### Separate vs joint comparison

The input signal is assumed to be Gaussian distributed and to have 1.56 effective number of bits per sample. Both joint coding and separate coding systems are compared using Samsung 28 nm technology. Specifically, the PCM array is an ‘8KB’ PCM array, with 256 rows x 256 columns, simulated using NVSIM. C++ code for this simulation is available at https://github.com/SEAL-UCSB/NVSim. In the separate coding system, the input is first converted by a 2-bit ADC into digital values (though in a more realistic scenario even higher precision ADC would likely be required). The digital values are then encoded by an LDPC encoder. The encoded signals are then written into the PCM array. In the separate coding scheme, each PCM cell can store 2 bits, and one write pulse is adequate to program the PCM cell into one of four states. Therefore, with a code-rate 0.5, each input sample is encoded into 4 bits (two for the signal and two for the required error correction) written over two PCM devices. When retrieving stored information, it is decoded by an LDPC decoder (energy efficiency statistics can be found in Table 2.3 of^45^) and finally converted back into analog form using a 2-bit DAC. This LDPC decoding step typically requires at least 10 iterations to converge. With such an implementation, the energy efficiency is ~128 pJ/sample. The most energy-consuming stage is PCM write (70pJ/sample) – necessitated by the redundant devices required for digital error correction – followed by LDPC decoding (44pJ/sample).

In the joint coding implementation, the input analog signal is first converted by an ADC into digital values. The values then pass through the encoding to determine the voltage to be applied to the PCM. As in the separate case, under the joint coding scheme each PCM cell can store 2 bits. However, in this case, as error correction is built in to the encoding and decoding functions, we do not require redundant, error-correcting bits. Therefore, one device is adequate to store one input sample (i.e. code rate = 1). The PCM is then read by an ADC of the same precision as the input ADC. The resulting values are transformed by the decoding function and finally converted back to an estimate of the analog signal by a DAC. Under such scheme, the energy efficiency is highly dependent on the required bit-precision of ADC/DACs. We used the ADC/DAC survey from^60^ and found that the energy per conversion (at 28 nm) scales roughly exponentially with the effective number of bits (Fig. M1). Therefore, the joint coding implementation offers more benefit when the required ADC/DAC precision is low (Fig. M2).

As the input signal has only 1.56 bits per/sample, a 4-bit ADC/DAC suffices for the joint coding scheme. If the input samples contained more information, a higher precision ADC/DAC would be required, eventually reaching the point where the separate scheme outperformed the joint. This is an experimental demonstration of the principle laid out by Rahul Sarpeshkar in^61^, namely that analog computation is efficient at low-precision, while digital becomes efficient at high-precision. Thus, analog representations are best in this case when the required precision is low to moderate (precisely the kinds of scenarios where the majority of the analog data being produced is used – e.g. human or statistical machine inference).

On a final note, as we are trying to make a comparison between analog and digital strategies, implementing the analog version using ADC/DAC might seem inappropriate. However, this can actually be seen as a sort of easily implementable “intermediate” between fully analog and fully digital, as this scheme is using the analog coding strategy – namely, the functions learned for encoding/decoding – but digitizing it for easy implementation with existing technology. That being said, a faithful analog implementation would perform even better than what we have presented here as it would remove the need for ADC/DAC. Specifically, this analysis could have been done by building custom op-amps to truly implement the analog functions. But this would have required extensive work (likely necessitating an additional paper for characterization and construction details) as comparatively less time has been spent on optimizing analog electronics due to the ubiquity of digital hardware.

## Supplementary information

Supplementary information.
