# Author Correction: Analog Coding in Emerging Memory Systems

**DOI:** 10.1038/s41598-020-70121-y

**Published:** 2020-08-04

**Authors:** Ryan V. Zarcone, Jesse H. Engel, S. Burc Eryilmaz, Weier Wan, SangBum Kim, Matthew BrightSky, Chung Lam, Hsiang-Lan Lung, Bruno A. Olshausen, H.-S. Philip Wong

**Affiliations:** 1 Redwood Center for Theoretical Neuroscience, UC Berkeley, Berkeley, CA 94720 US; 2 Department of Electrical Engineering and Stanford SystemX Alliance, Stanford University, Stanford, CA 94305 US; 3 IBM Research, T.J. Watson Research Center, Yorktown Heights, NY 10598 US; 4 Macronix International Co., Ltd., Emerging Central Lab, 16 Li-Hsin Road, Hsinchu Science Park, Taiwan; 5 Google Brain, 1965 Charleston Rd., Mountain View, CA 94043 US

Correction to: *Scientific Reports*https://doi.org/10.1038/s41598-020-63723-z, published online 22 April 2020

The Acknowledgements section in this Article is incomplete.

“This work was supported in part by SONIC, one of six centers of STARnet, a Semiconductor Research Corporation program sponsored by MARCO and DARPA, by member companies of the Stanford SystemX Alliance, and member companies of the Stanford Non-Volatile Memory Technology Research Initiative (NMTRI). R.Z. was supported by a National Science Foundation Graduate Research Fellowship under Grant No. DGE 1752814”.

should read:

“This work was supported in part by SONIC, one of six centers of STARnet, a Semiconductor Research Corporation program sponsored by MARCO and DARPA, by member companies of the Stanford SystemX Alliance, member companies of the Stanford Non-Volatile Memory Technology Research Initiative (NMTRI), and a research gift from Intel Corporation. R.Z. was supported by a National Science Foundation Graduate Research Fellowship under Grant No. DGE 1752814”.

