# Supplementary material for: Analog Coding in Emerging Memory Systems
Source: Sci Rep. 2020 Apr 22;10:6831. doi: 10.1038/s41598-020-63723-z (PMC7176644; doi:10.1038/s41598-020-63723-z)
Supplement: Supplementary file 1 — Supplementary information. [file 41598_2020_63723_MOESM1_ESM.pdf]

[Supplemental] Analog Coding in Emerging Memory Systems

Ryan V. Zarcone[1*†], Jesse H. Engel[1,2,5,*†], S. Burc Eryilmaz[2], Weier Wan[2], SangBum Kim[3], Matthew BrightSky[3], Chung Lam[3], Hsiang-Lan Lung[4], Bruno A. Olshausen[1], and H.-S. Philip Wong[2]

[1] Redwood Center for Theoretical Neuroscience, UC Berkeley, Berkeley, CA, 94720
[2] Dept. of Electrical Engineering and Stanford SystemX Alliance, Stanford University, Stanford, CA, 94305
[3] IBM Research, T.J. Watson Research Center, Yorktown Heights, NY, 10598
[4] Macronix International Co., Ltd., Emerging Central Lab, 16 Li-Hsin Road, Hsinchu Science Park, Taiwan
[5] Google Brain, 1965 Charleston Rd., Mountain View, CA, 94043
[*] Email: {ryan.zarcone, jesse.engel}@gmail.com
[†] Denotes equal contribution

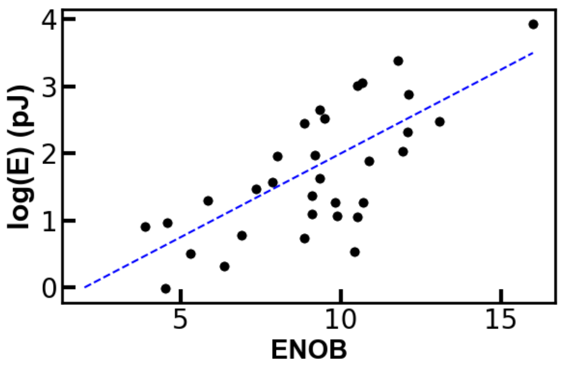

Figure M1: Scaling of ADC/DAC energy per conversion (pJ) vs. effective number of bits (ENOB) at 28nm. Numbers taken from the Stanford ADC/DAC performance survey [60]. Energy per conversion scales approximately exponentially with the number of effective bits. Dashed line indicates fit with $y \cong 10^{x/4}$

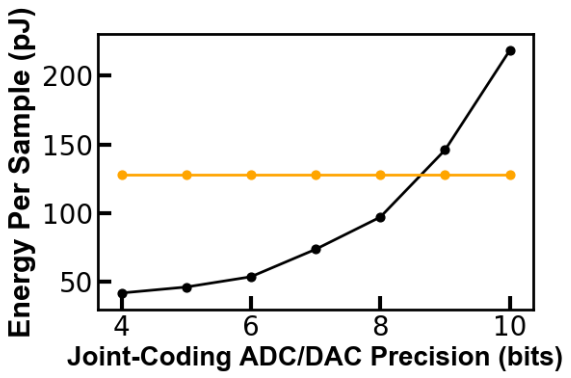

Figure M2: Energy efficiency of joint coding systems vs Separate coding system at different joint coding ADC/DAC precisions. Using the simulated 8KB array, we estimate the energy per sample required for the joint coding system at different joint coding ADC/DAC precisions (black line) while keeping the precision of the separate scheme fixed to its lowest possible value for the task (orange line). The energy per sample for joint coding scales exponentially, and up until approximately 8.5 bits of precision, the joint scheme is more efficient. A joint coding approach is, therefore, more efficient for tasks when the required precision is low to moderate (e.g. object detection in images).
